# X-linked Bilateral Periventricular Nodular Heterotopia

**DOI:** 10.5334/jbsr.2086

**Published:** 2020-05-06

**Authors:** Yannick Thonissen, Luc De Catte, Michael Aertsen

**Affiliations:** 1 University Hospitals Leuven, BE

**Keywords:** Fetal imaging, post-mortem imaging, genetic disorders, neuronal migration disorders

## Abstract

**Teaching Point**: Postmortem magnetic resonance imaging can replace conventional autopsy and help diagnosis in combination with other postmortem investigations (e.g. microscopic examination of the placenta, genetic testing, etc.) under the umbrella of minimal invasive autopsy.

## Case History

A 26-year-old patient was referred at 25 weeks of gestation to our tertiary center for further diagnostic work-up after visualisation of a mega cisterna magna on routine ultrasound at a gestational age (GA) of 20 weeks. Neither one of the parents, nor the first-born daughter have a significant medical history.

**Figure 1:**
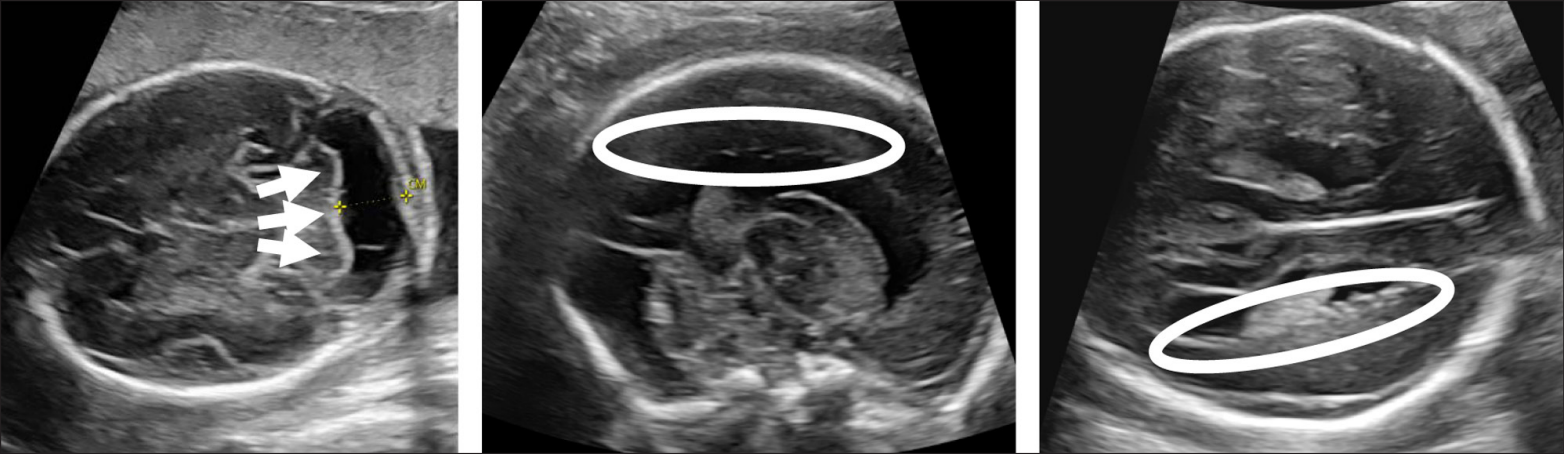
Prenatal ultrasound images at 25 weeks of gestation demonstrate a dilatation of the cisterna magna >10 mm (arrows) and nodular lining of walls of the lateral ventricles (oval).

**Figure 2:**
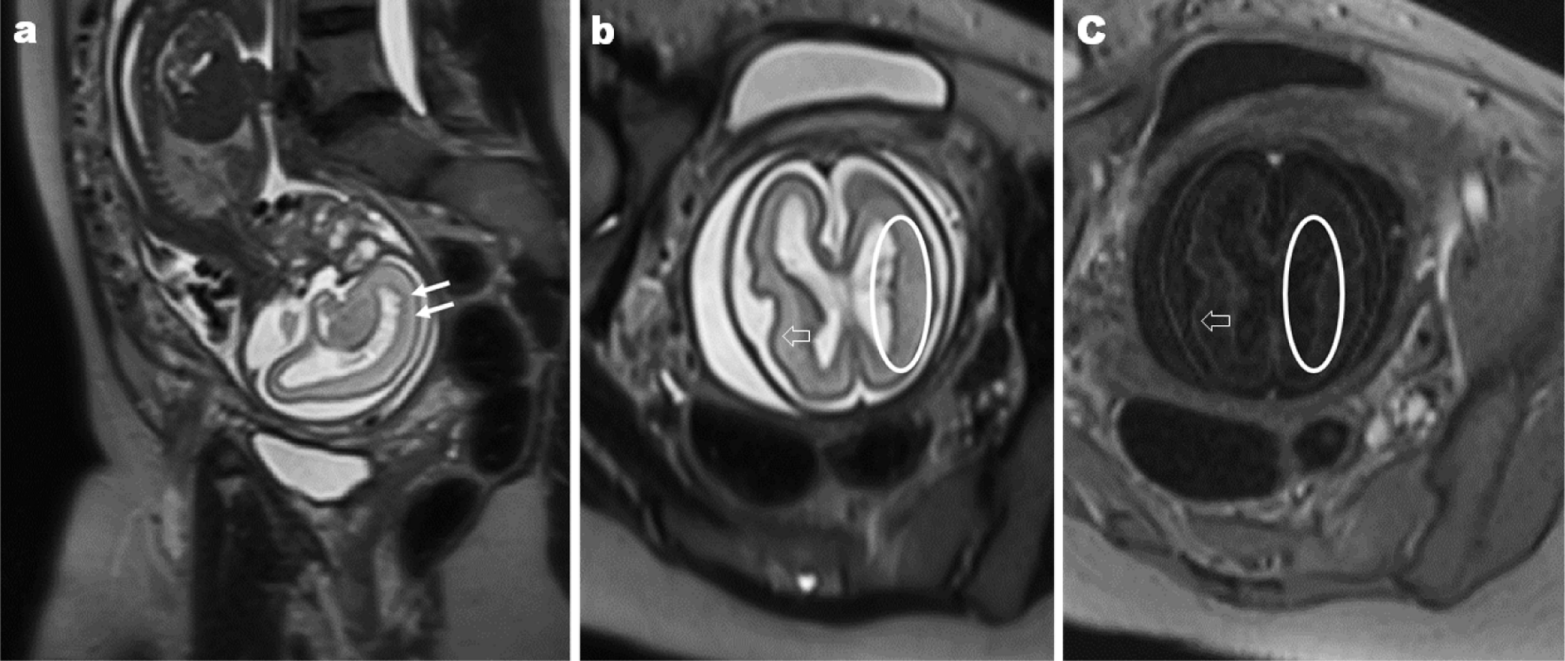
Sagittal and axial T2w images **(a, b)** and axial T1w image **(c)** of the brain performed at a gestational age of 25 3/7 weeks. The ventricular lining (closed arrows and ovals) shows a bilateral nodular thickening following the signal intensities of the grey matter (open arrows).

An expert neurosonogram (Figure 1) at 25 weeks of GA demonstrated enlargement of the cisterna magna (thick arrows; 12 mm, normally <10 mm). Furthermore, an irregular lining of the lateral ventricles was noted on the axial and sagittal sections (ovals). Fetal MRI (Figure 2), performed three days later, confirmed the previously described irregular lining of the lateral ventricles (ovals and thin arrows) following the grey matter on T2- and T1-weighted images (open arrows) and enlarged cisterna magna. No additional anomalies were detected.

**Figure 3:**
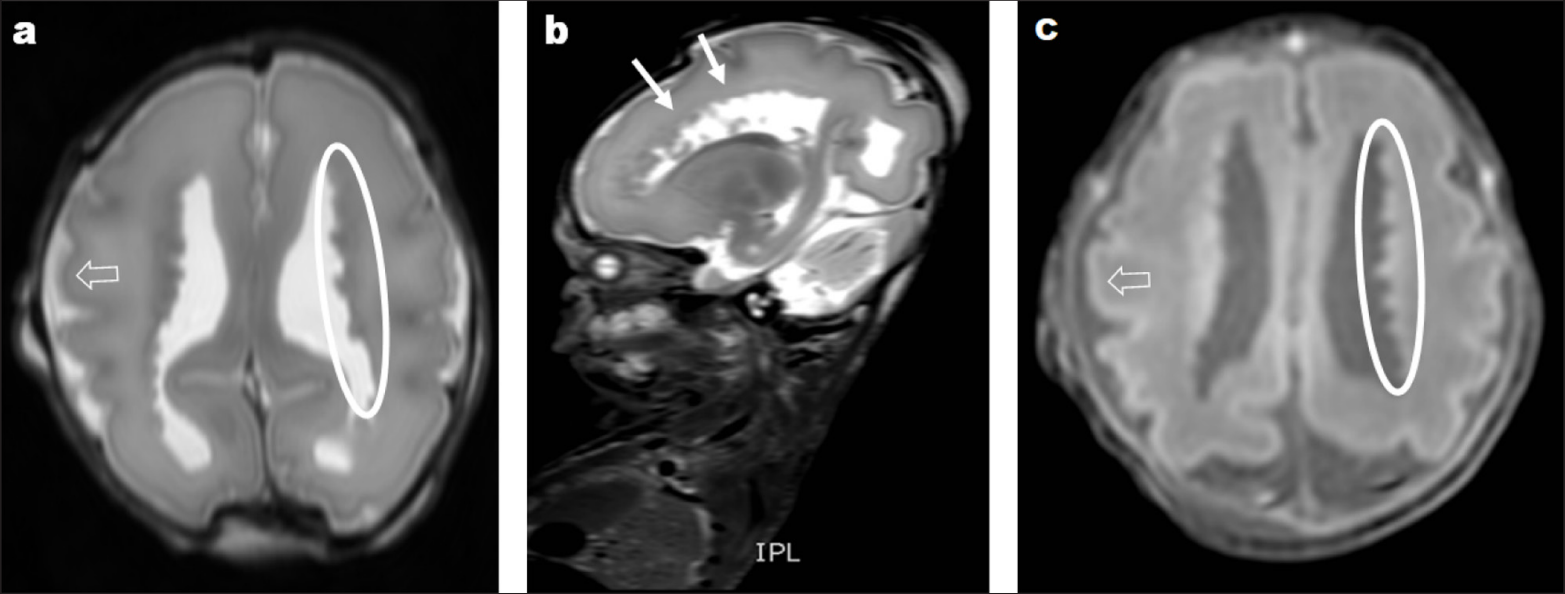
Post-mortem axial and sagittal T2w **(a, b)** and axial T1w **(c)** MR images of the brain at a fetal age of 27 weeks. The ventricles are lined with multiple nodules (closed arrows and ovals) following the signal intensities of the grey matter (open arrows).

A multidisciplinary team approved the couple’s request for termination of pregnancy. A post-mortem MRI was performed at an age of 27 weeks and 6 days (Figure 3), within 2 hours after delivery. This examination clearly showed the periventricular nodules (closed arrows and ovals) with signal intensities following the grey matter (open arrows) on T2- and T1-weighted images, predominantly located next to the frontal horns and ventricular bodies on both sides. No additional anomalies were detected.

Genetic testing showed a heterozygous deletion of the FLNA gene in both the mother and the fetus.

## Comment

Filamin A is a F-actin binding cytoplasmic phosphoprotein of huge importance in the neural migration. The encoding FLNA gene is located at Xq28 and loss of function mutation in the FLNA gene causes disorders of neural migration. The classic FLNA pattern comprises bilateral, periventricular heterotopic grey matter nodules (BPNH), mainly centered around the frontal horns and body of the lateral ventricles. Additional intracranial findings may include a dysplastic corpus callosum or certain posterior fossa anomalies. When hippocampal malformations, cerebellar hypoplasia, polymicrogyria or microcephaly are present, other etiologies are more likely [1].

The classical pattern is highly associated with a FLNA gene mutation, particularly in females. One study showed the mutation in 49% of classical BPNH regardless of sex, but a high 77% correlation in female patients. This gender bias can be explained by the high lethality in male fetuses [1].

The overall penetrance is not clearly established, but the MRI and clinical phenotypes are highly variable, probably due to skewed X-inactivation. Seizures varying in severity and age of onset are the main clinical features. There appears to be no correlation between imaging and clinical severity. Furthermore, this genetic mutation is associated with cardiovascular anomalies. As in our case, affected mothers without any clinical symptoms have been reported in the literature [1].

## Additional File

The additional file for this article can be found as follows:

10.5334/jbsr.2086.s1 X-linked BPNH. This case was previously presented during the Section Meeting Pediatric Radiology held by the BSR in June 2018. I would like to present you the slides for a more detailled and schematic overview of the disease.
