# Supplementary material for: X-linked Bilateral Periventricular Nodular Heterotopia
Source: J Belg Soc Radiol. 2020 May 6;104(1):23. doi: 10.5334/jbsr.2086 (PMC7207253; doi:10.5334/jbsr.2086)
Supplement: X-linked BPNH. — This case was previously presented during the Section Meeting Pediatric Radiology held by the BSR in June 2018. I would like to present you the slides for a more detailled and schematic overview of the disease. [file jbsr-104-1-2086-s1.PPTX]

## Slide 1
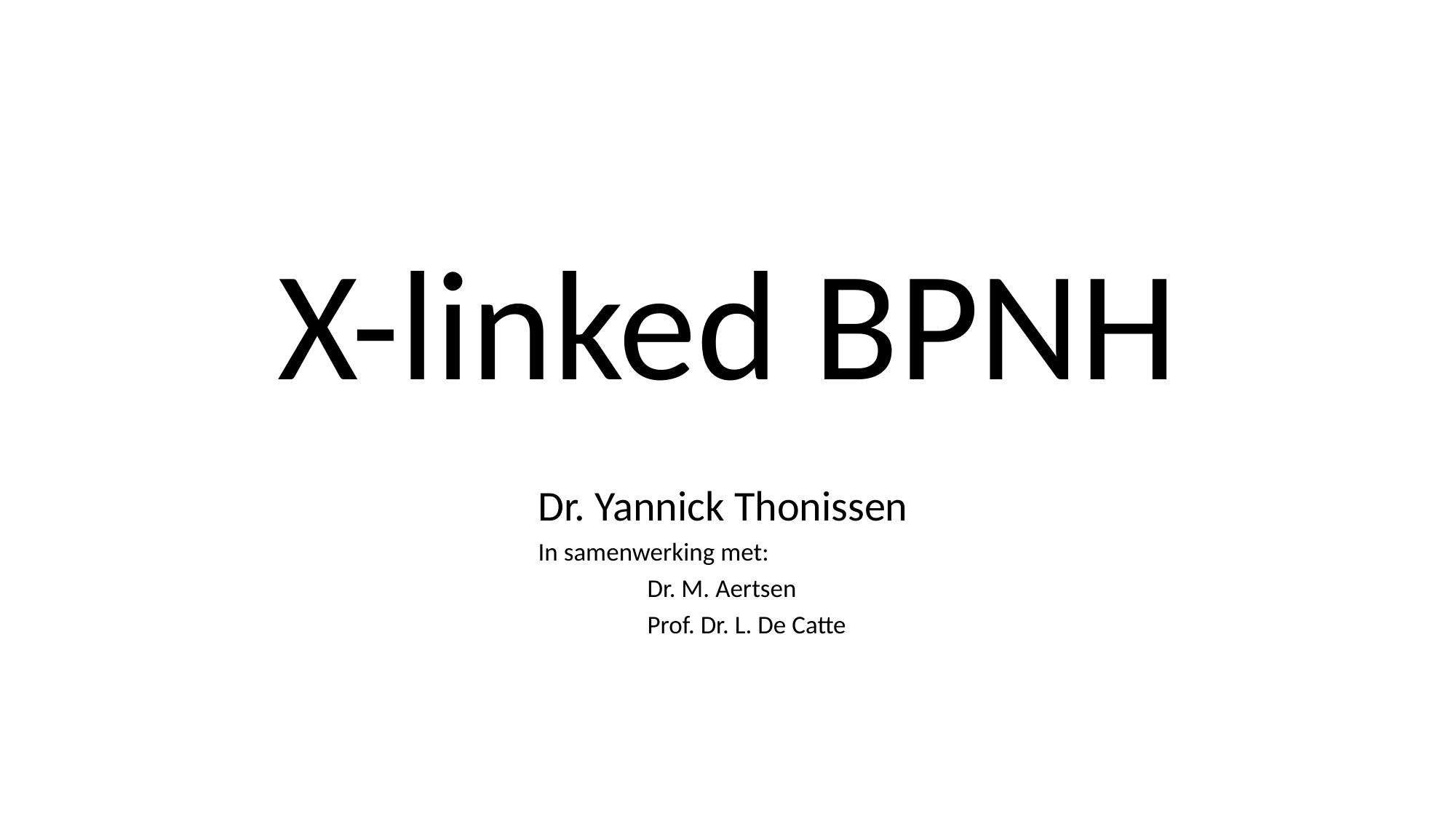

# X-linked BPNH
Dr. Yannick Thonissen
In samenwerking met:
	Dr. M. Aertsen
	Prof. Dr. L. De Catte

## Slide 2
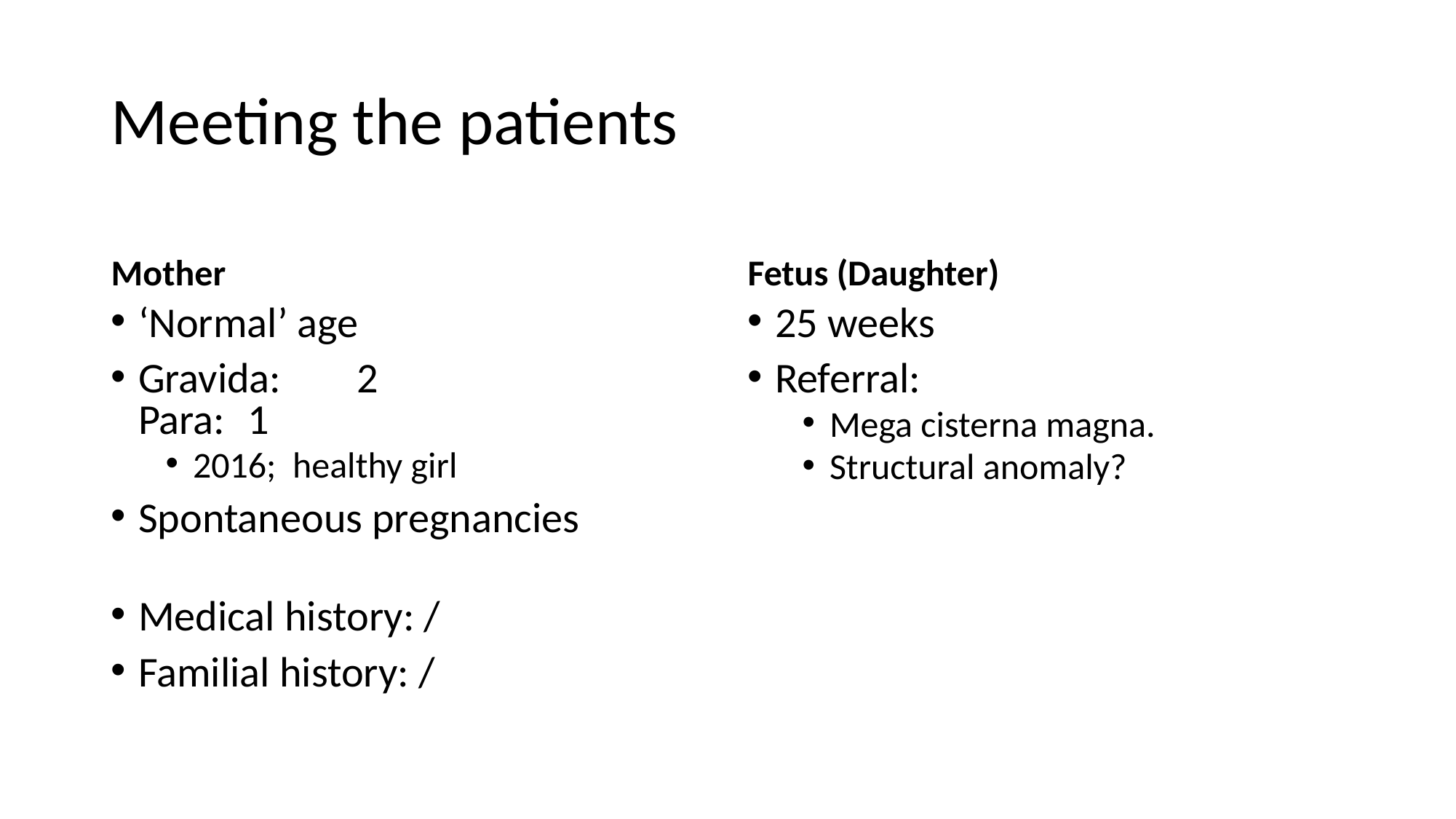

# Meeting the patients
Mother
Fetus (Daughter)
‘Normal’ age
Gravida: 	2
Para: 	1
2016; healthy girl
Spontaneous pregnancies
Medical history: /
Familial history: /
25 weeks
Referral:
Mega cisterna magna.
Structural anomaly?

## Slide 3
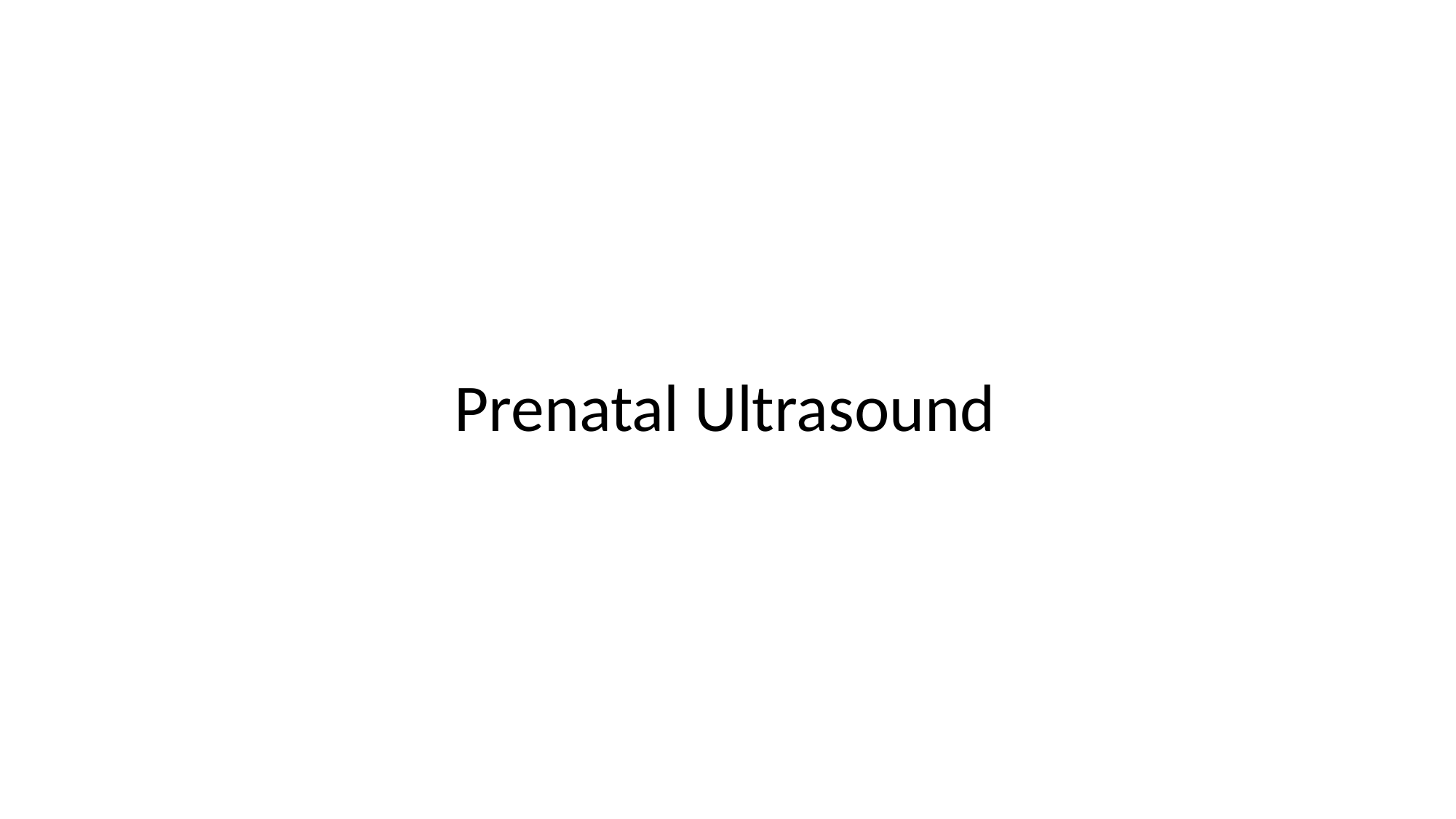

# Prenatal Ultrasound

## Slide 4
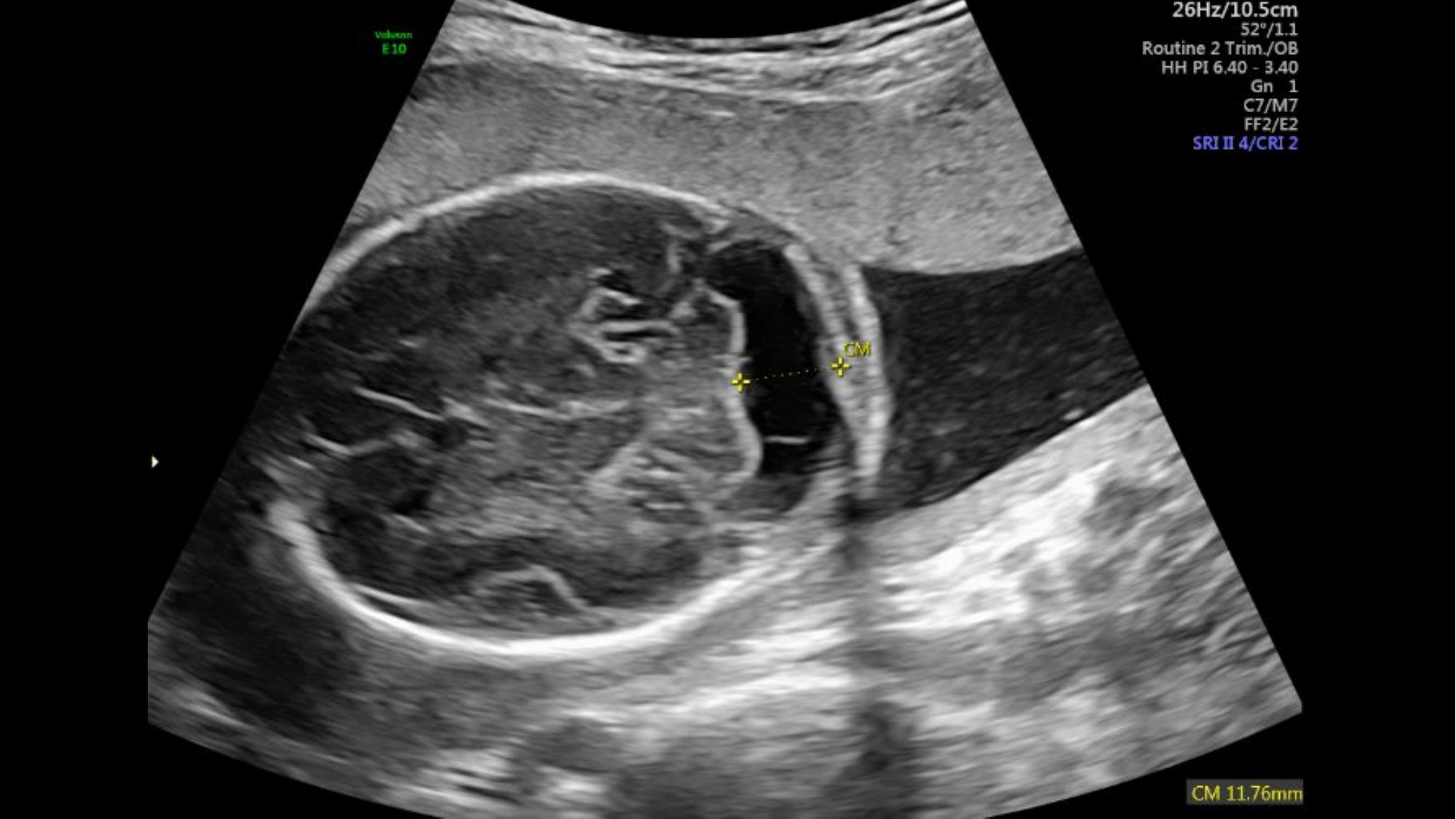

## Slide 5
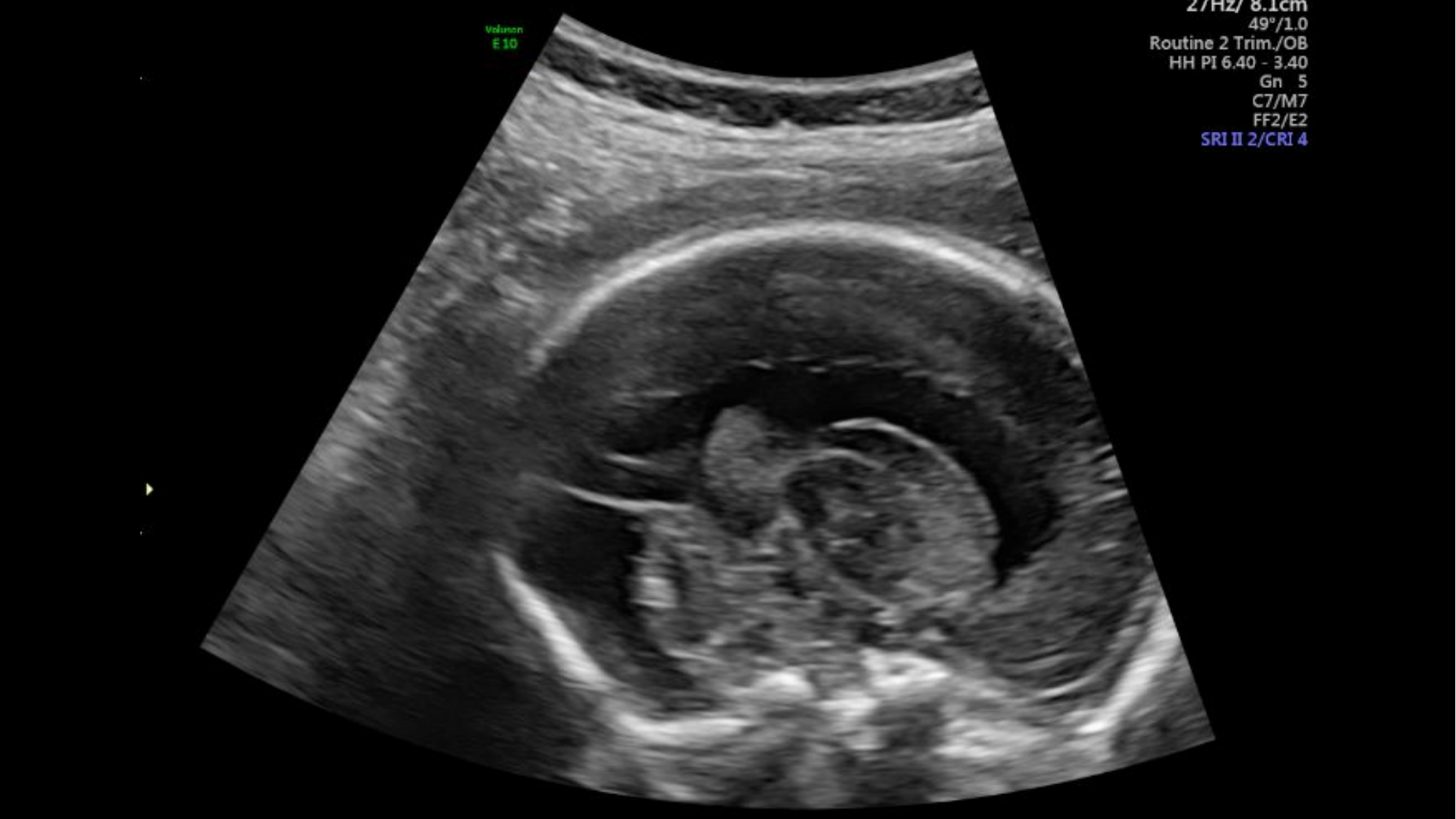

## Slide 6
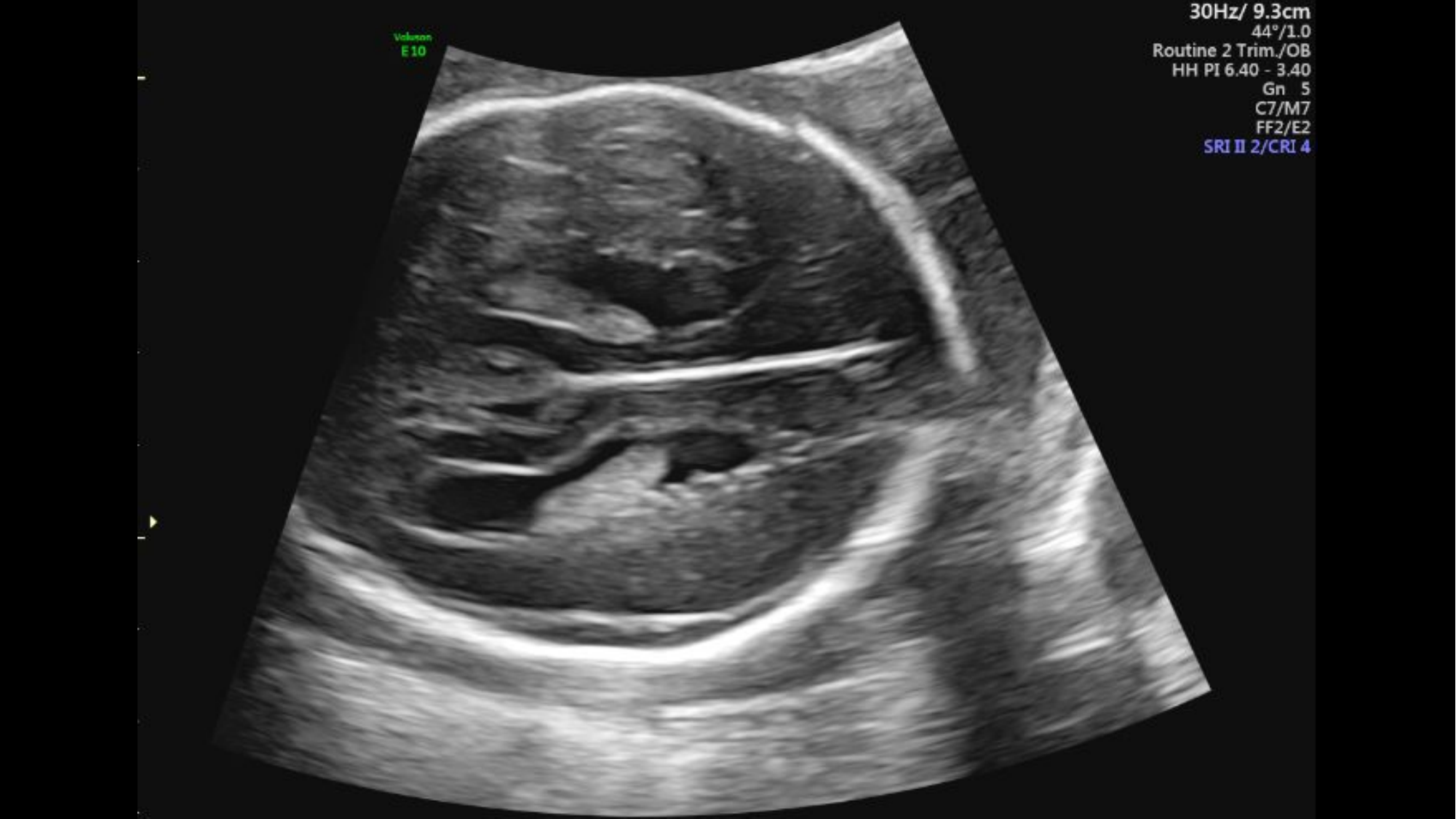

## Slide 7
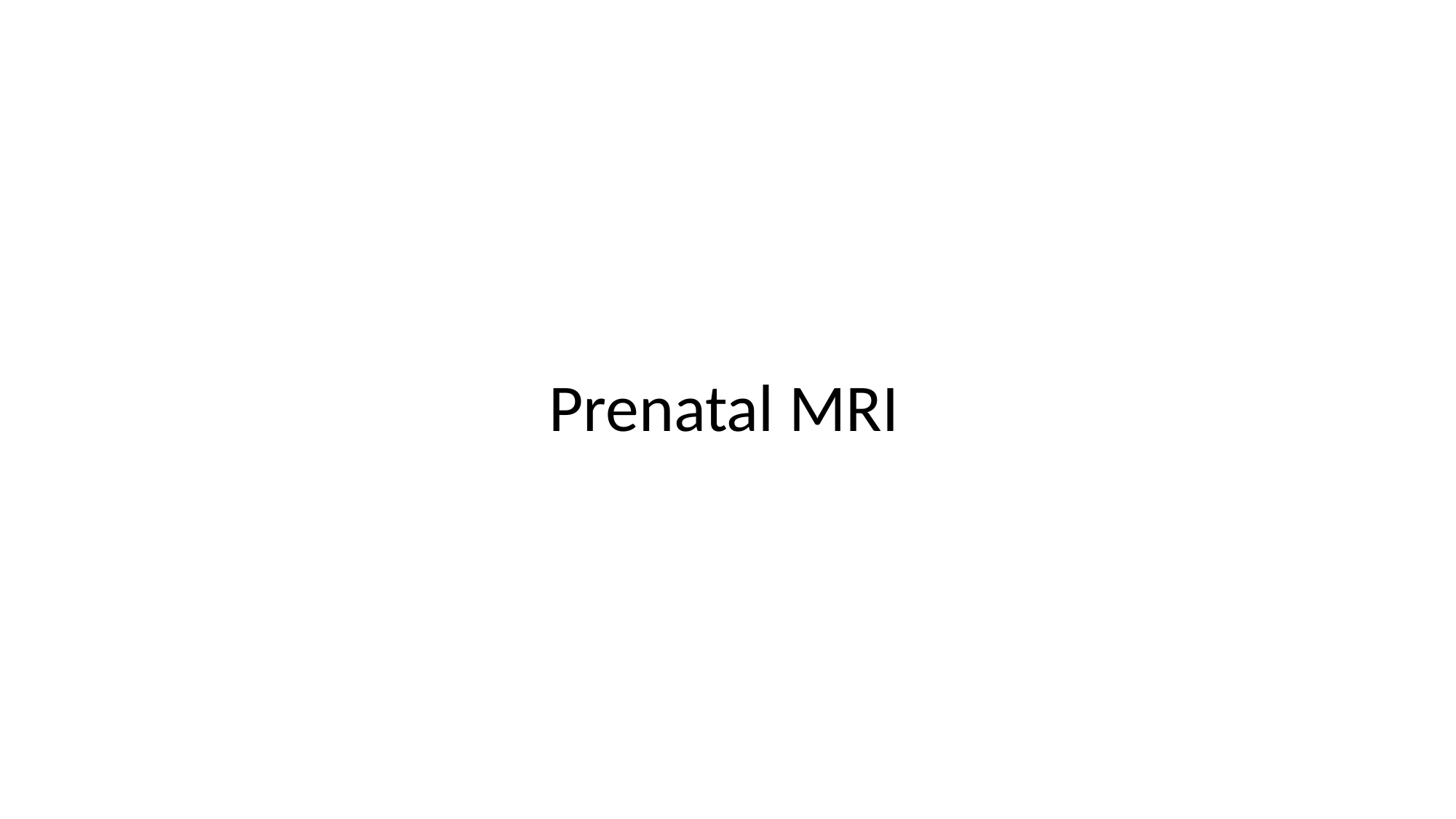

# Prenatal MRI

## Slide 8
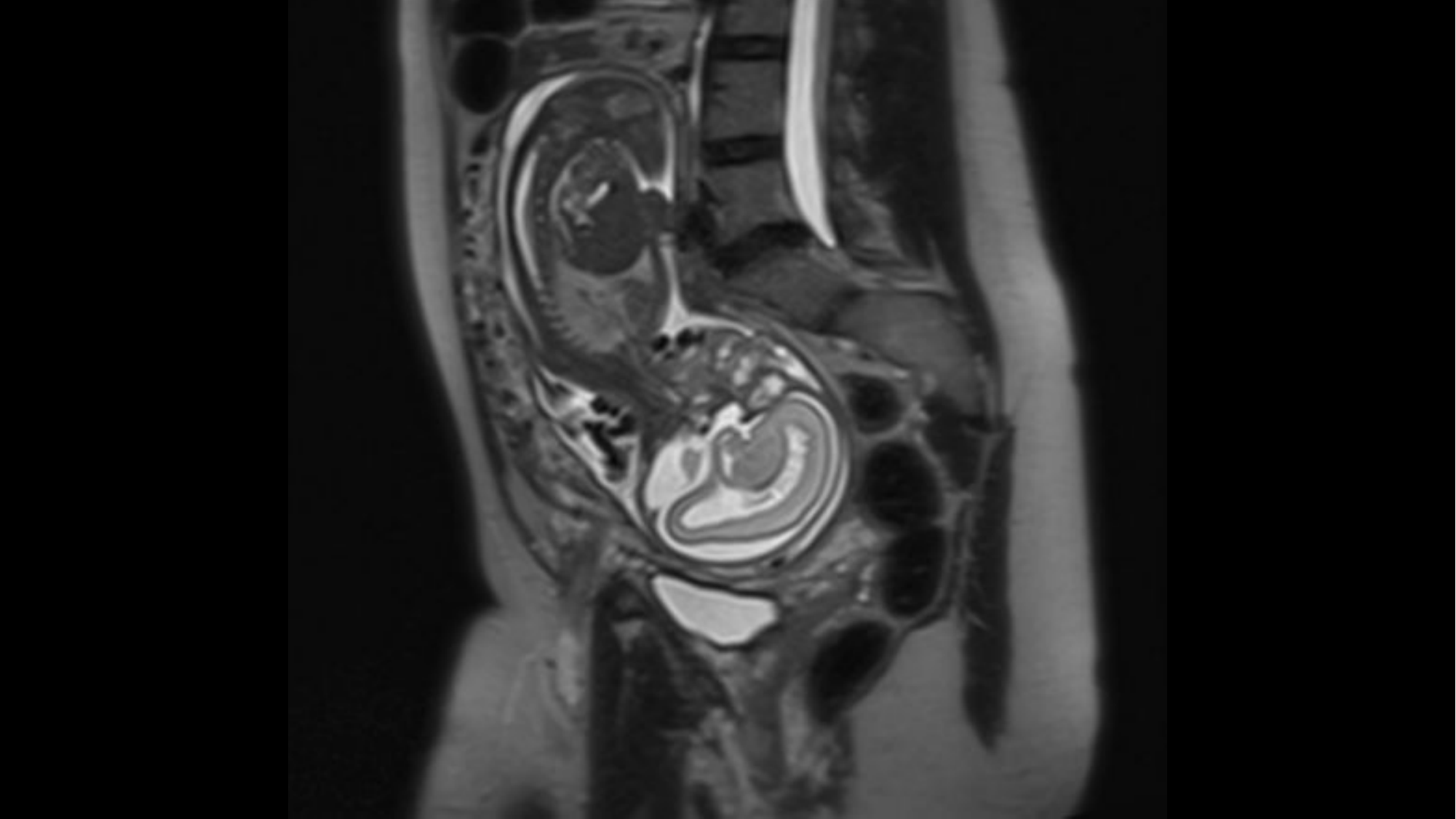

## Slide 9
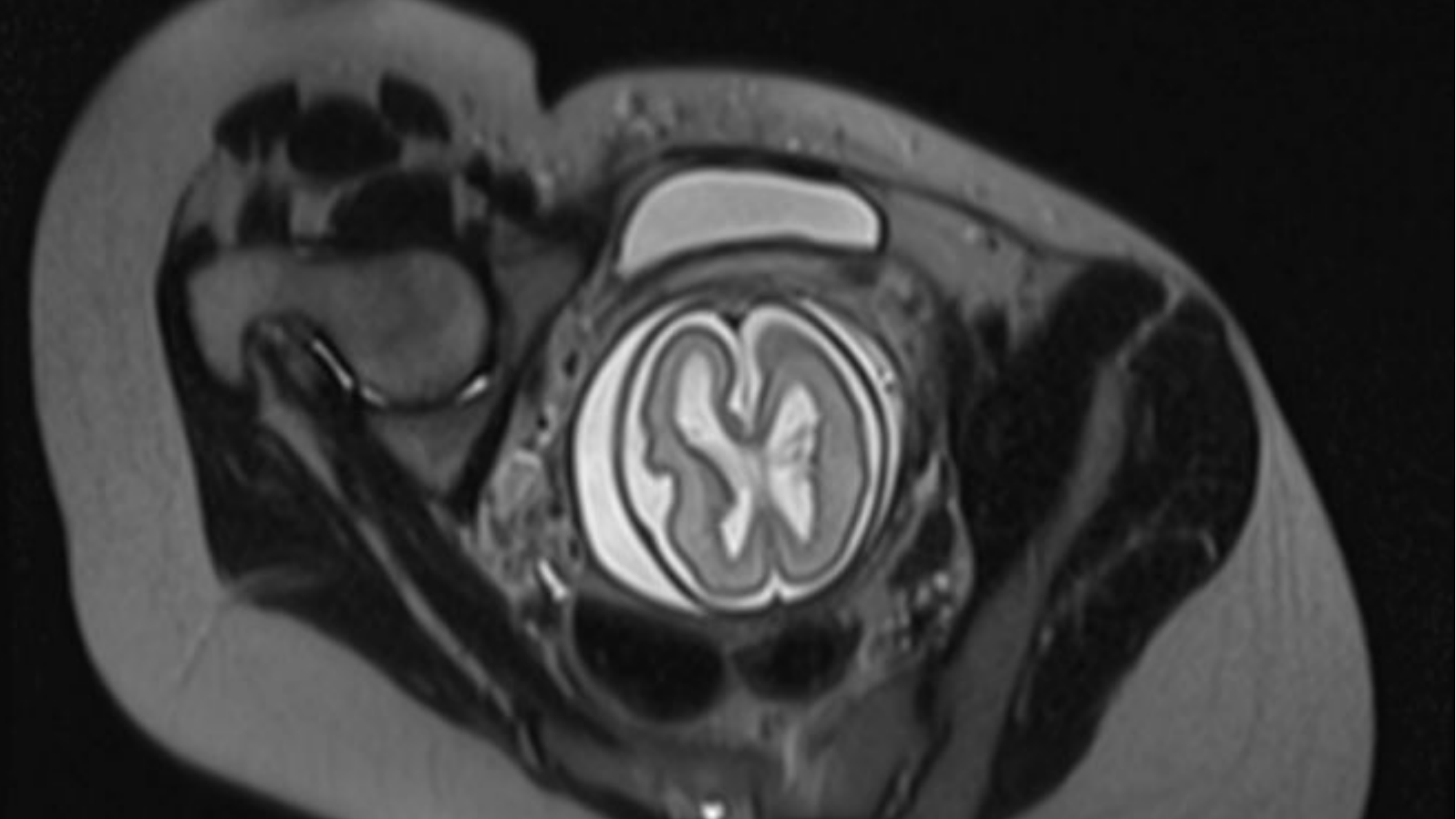

## Slide 10
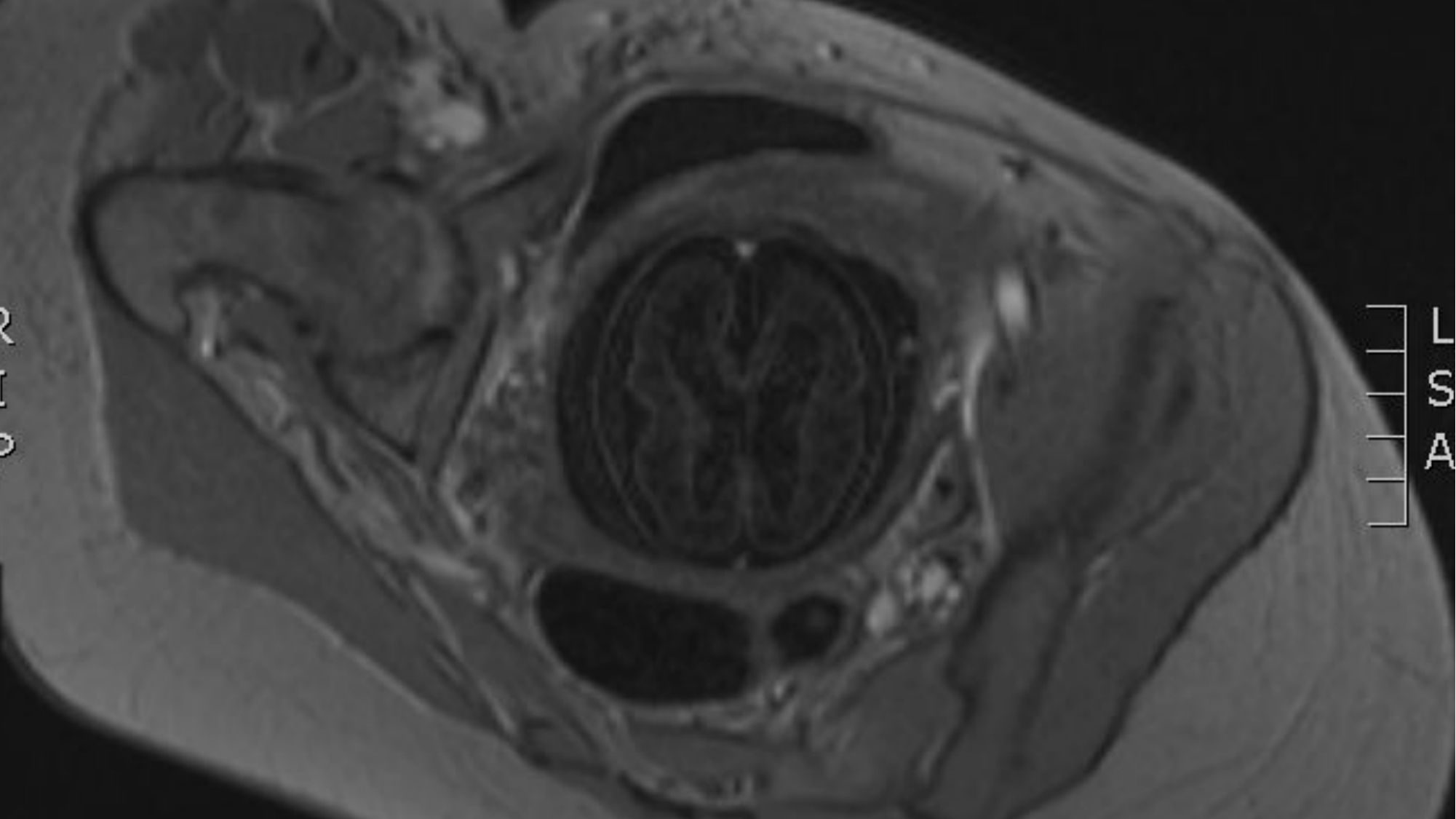

## Slide 11
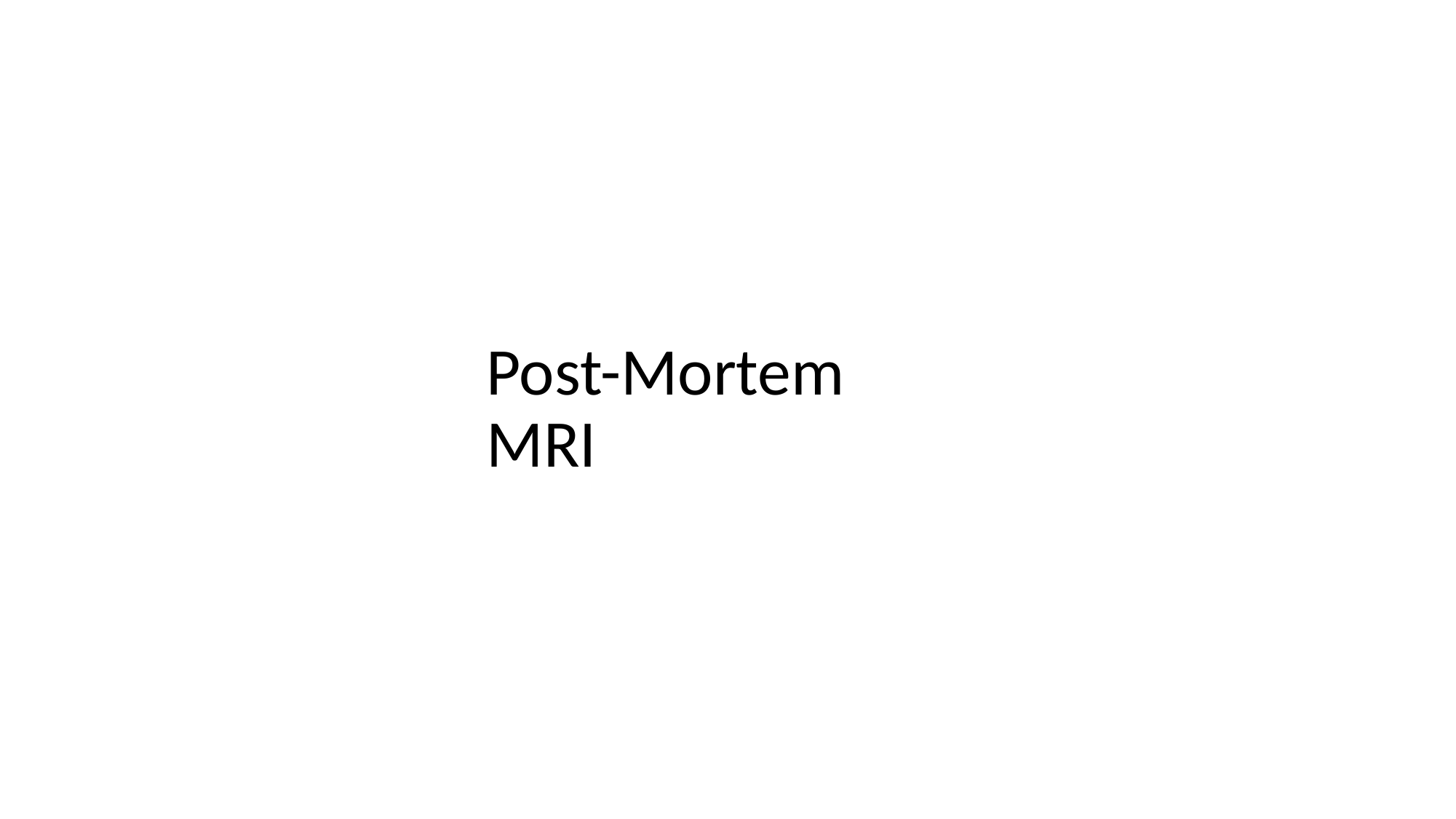

# Post-Mortem MRI

## Slide 12
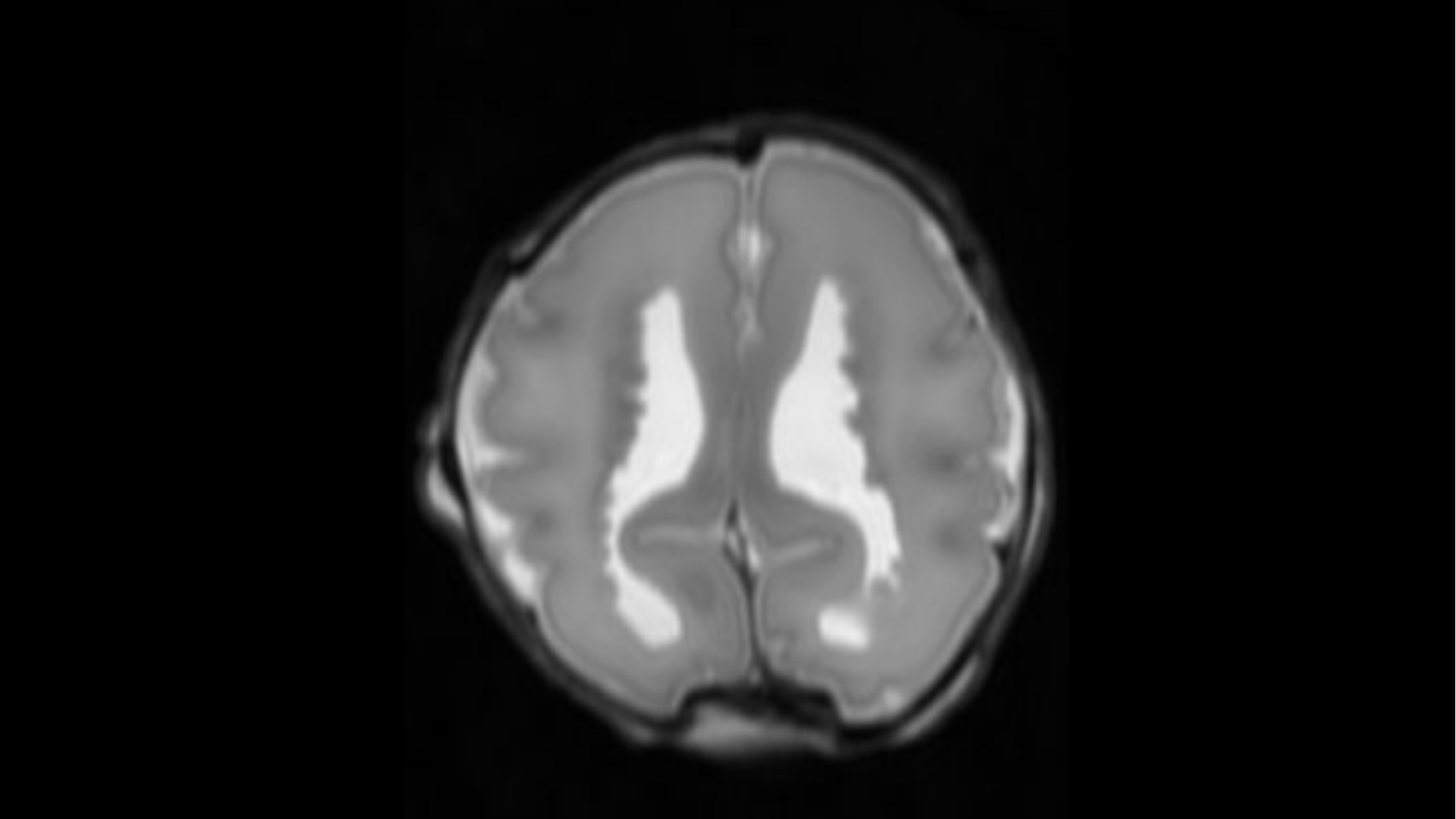

## Slide 13
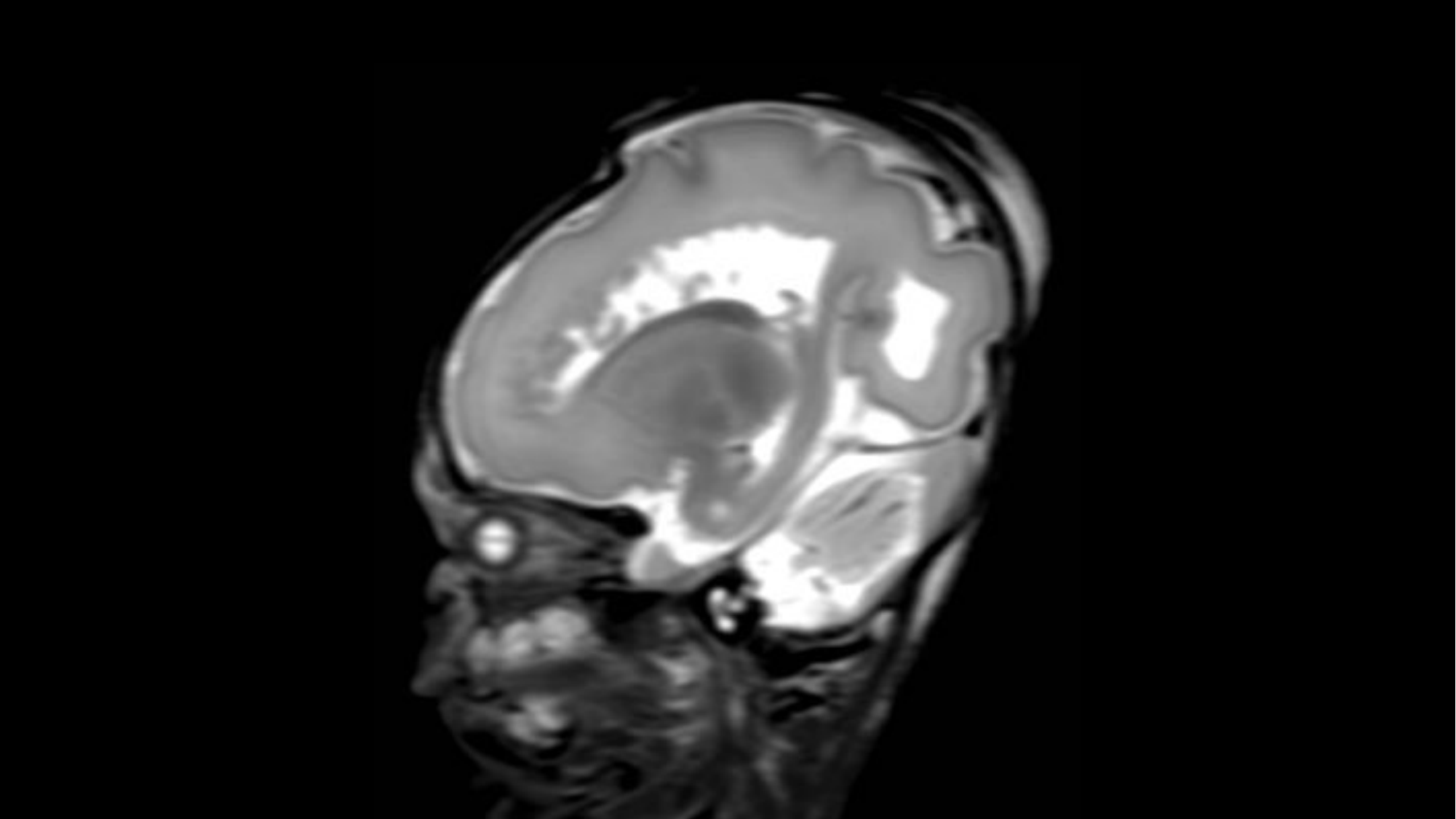

## Slide 14
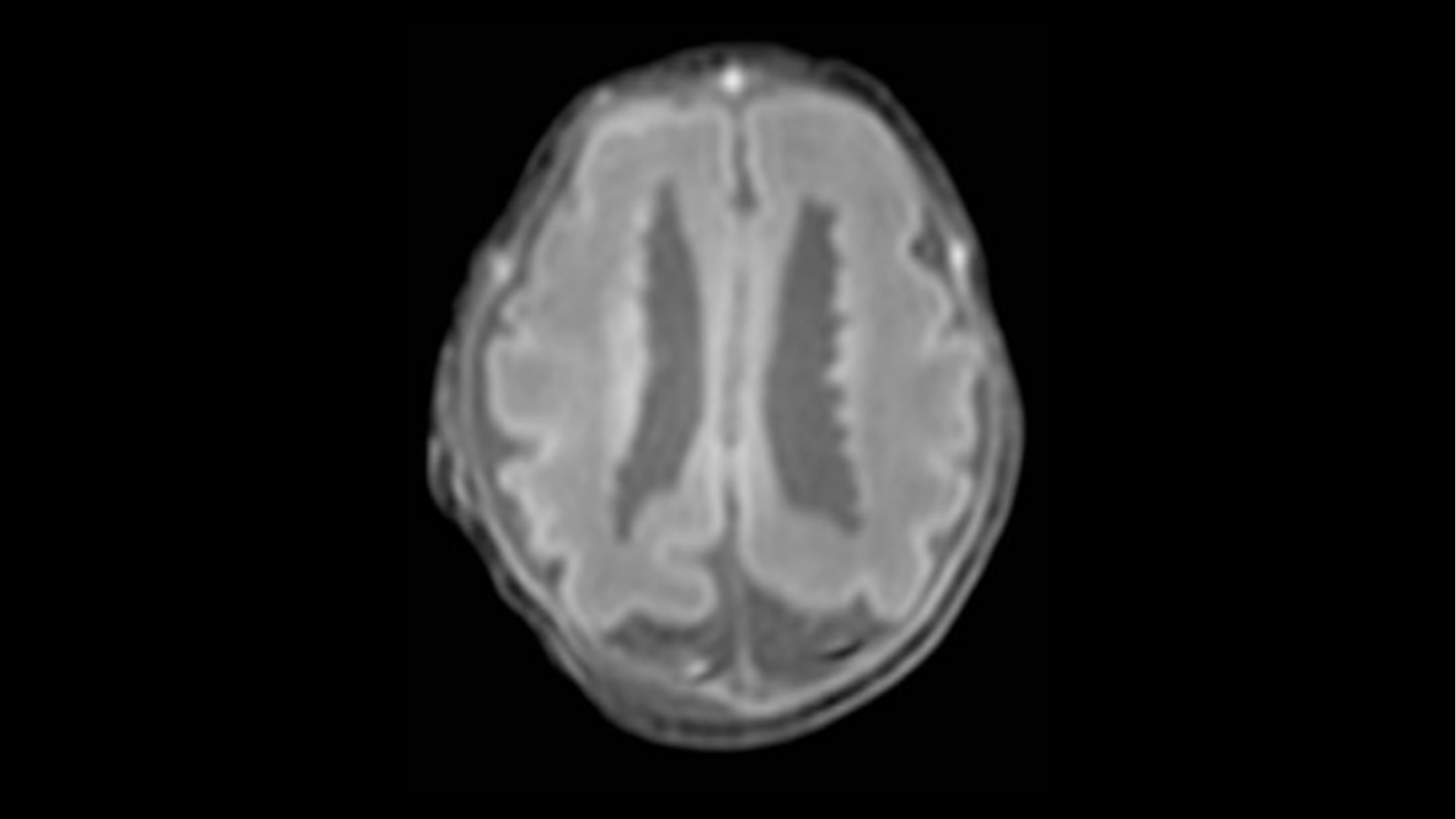

## Slide 15
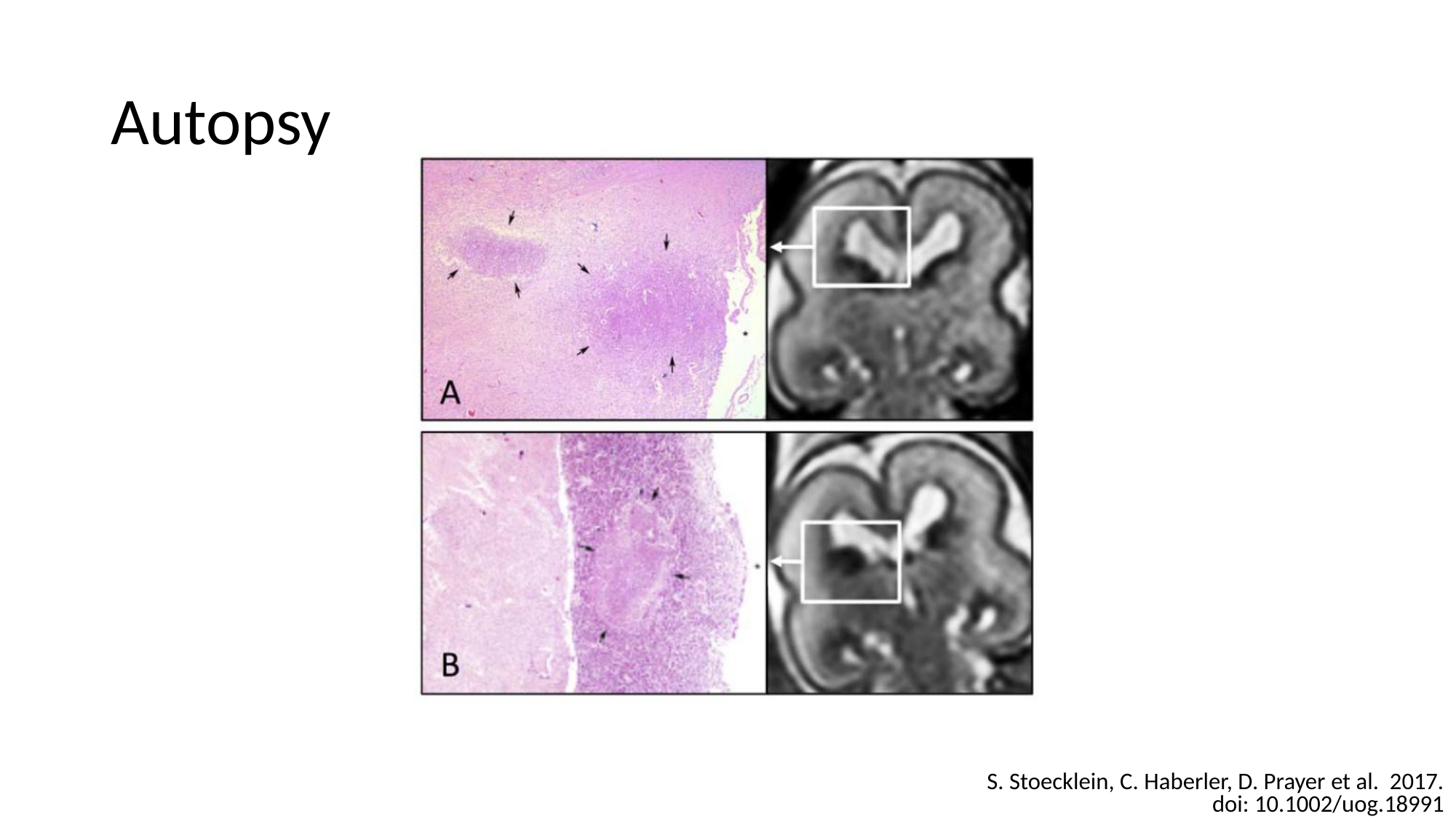

# Autopsy
S. Stoecklein, C. Haberler, D. Prayer et al. 2017.
doi: 10.1002/uog.18991

## Slide 16
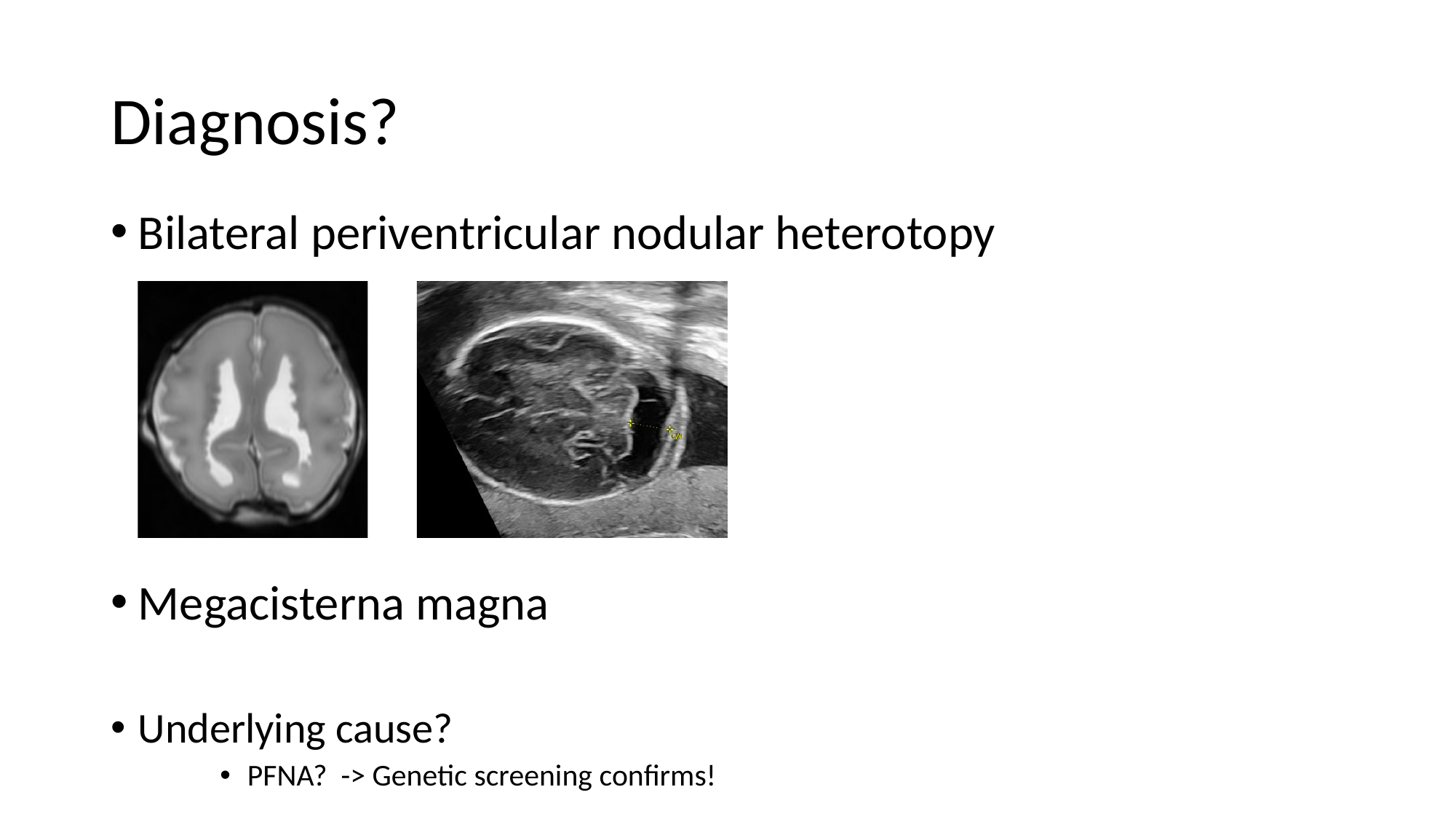

# Diagnosis?
Bilateral periventricular nodular heterotopy
Megacisterna magna
Underlying cause?
PFNA? -> Genetic screening confirms!

## Slide 17
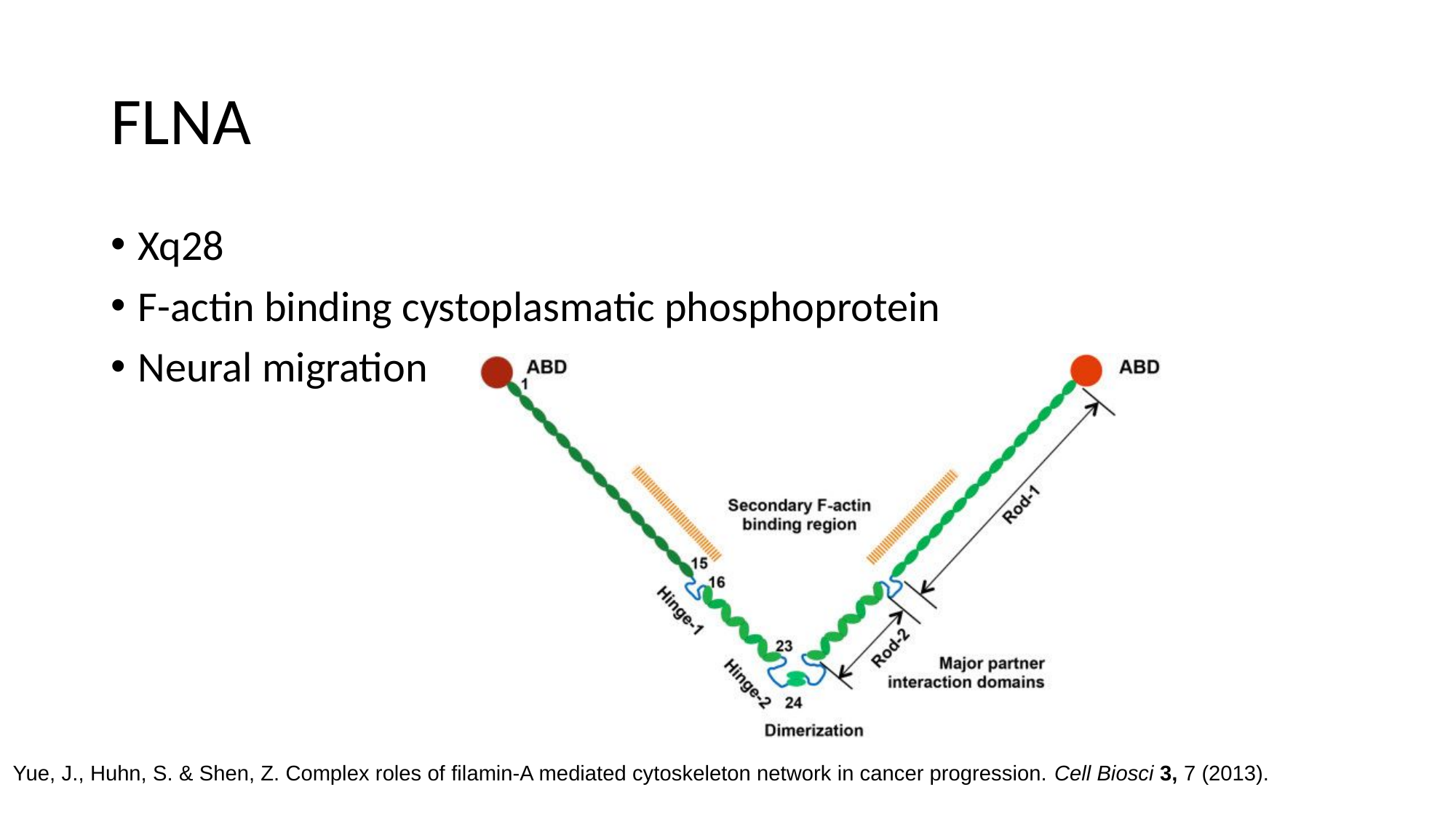

# FLNA
Xq28
F-actin binding cystoplasmatic phosphoprotein
Neural migration
Yue, J., Huhn, S. & Shen, Z. Complex roles of filamin-A mediated cytoskeleton network in cancer progression. Cell Biosci 3, 7 (2013).

## Slide 18
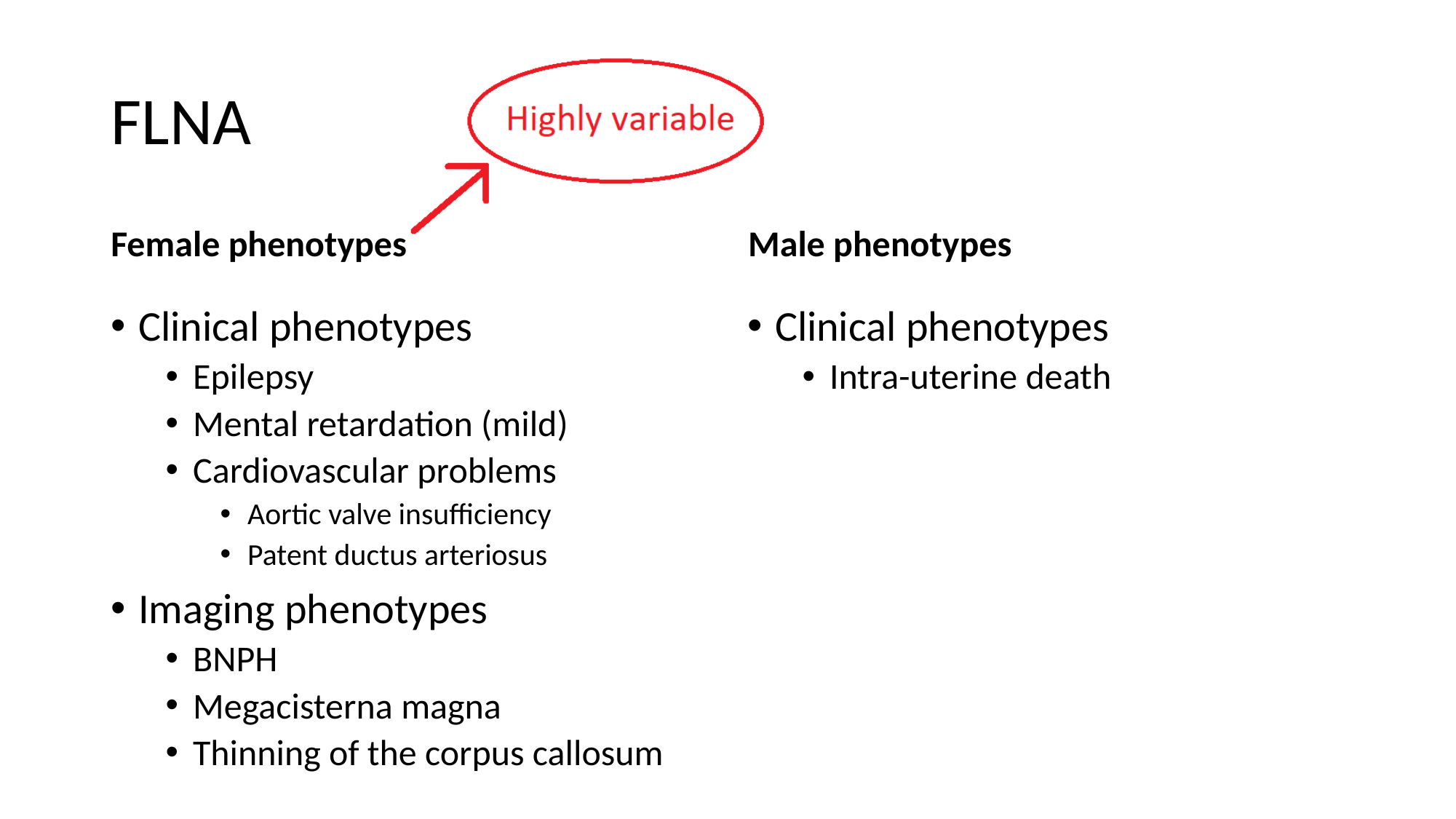

# FLNA
Female phenotypes
Male phenotypes
Clinical phenotypes
Epilepsy
Mental retardation (mild)
Cardiovascular problems
Aortic valve insufficiency
Patent ductus arteriosus
Imaging phenotypes
BNPH
Megacisterna magna
Thinning of the corpus callosum
Clinical phenotypes
Intra-uterine death

## Slide 19
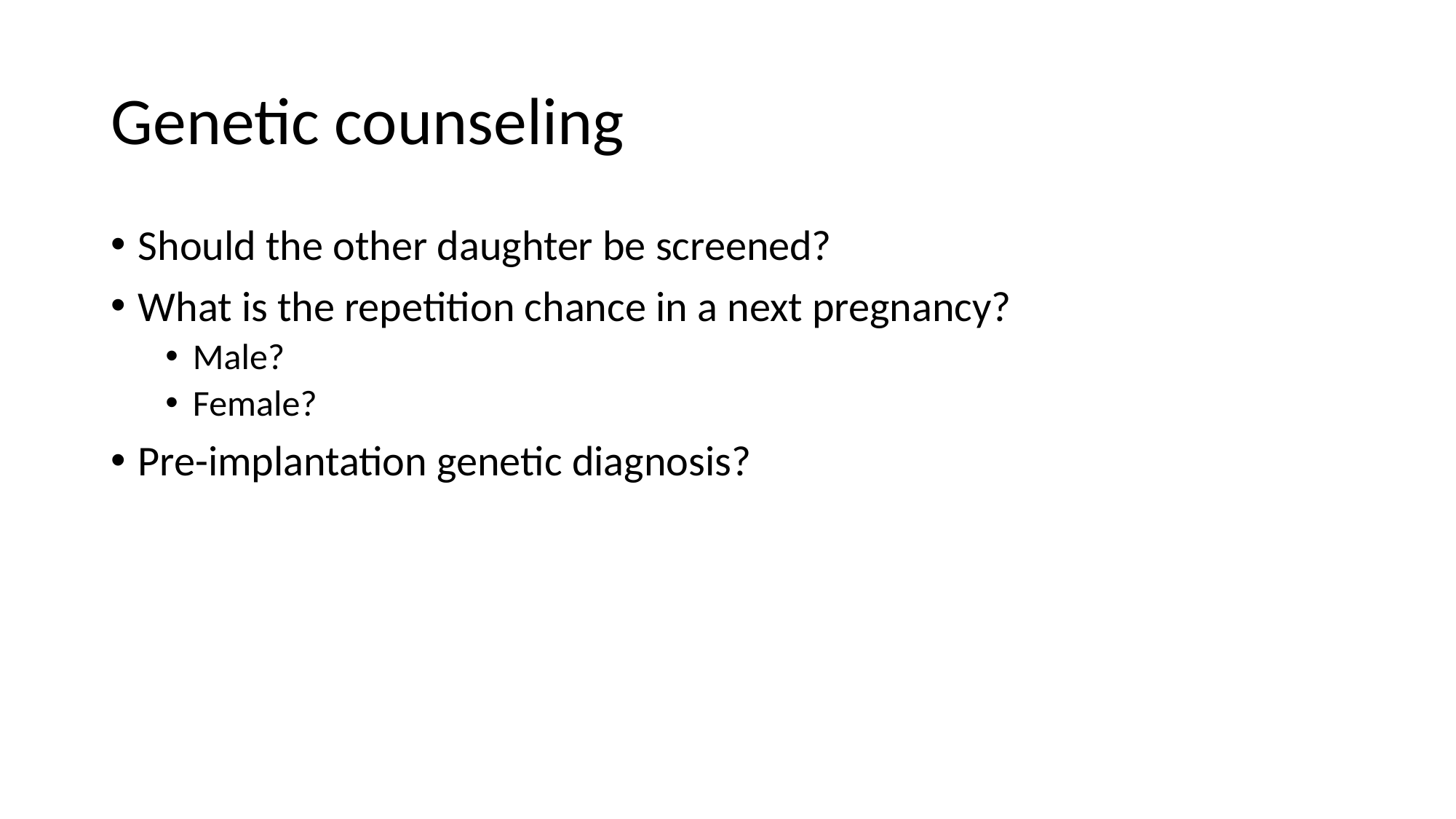

# Genetic counseling
Should the other daughter be screened?
What is the repetition chance in a next pregnancy?
Male?
Female?
Pre-implantation genetic diagnosis?

## Slide 20
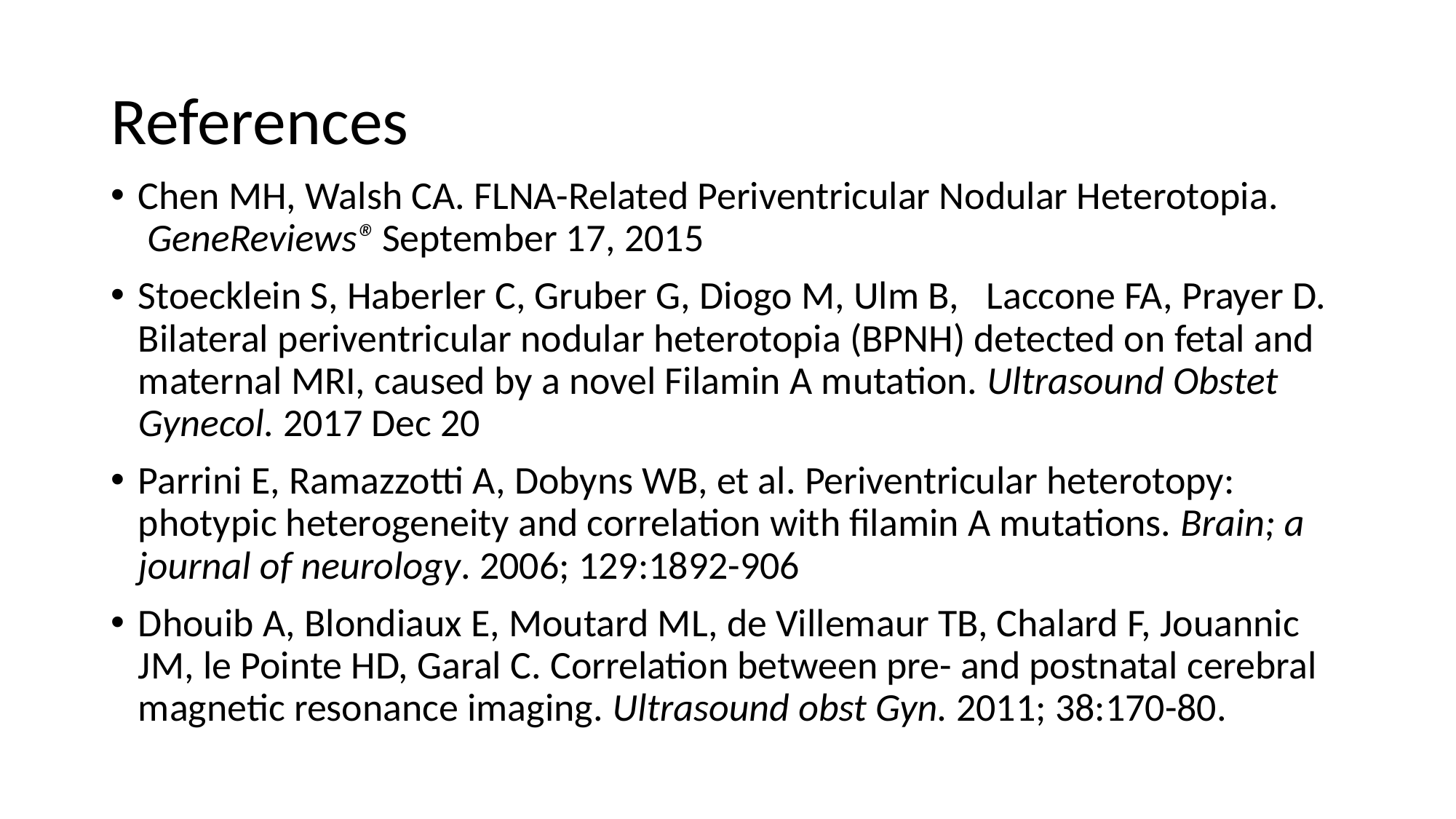

# References
Chen MH, Walsh CA. FLNA-Related Periventricular Nodular Heterotopia.  GeneReviews® September 17, 2015
Stoecklein S, Haberler C, Gruber G, Diogo M, Ulm B,  Laccone FA, Prayer D. Bilateral periventricular nodular heterotopia (BPNH) detected on fetal and maternal MRI, caused by a novel Filamin A mutation. Ultrasound Obstet Gynecol. 2017 Dec 20
Parrini E, Ramazzotti A, Dobyns WB, et al. Periventricular heterotopy: photypic heterogeneity and correlation with filamin A mutations. Brain; a journal of neurology. 2006; 129:1892-906
Dhouib A, Blondiaux E, Moutard ML, de Villemaur TB, Chalard F, Jouannic JM, le Pointe HD, Garal C. Correlation between pre- and postnatal cerebral magnetic resonance imaging. Ultrasound obst Gyn. 2011; 38:170-80.
